# Clinical Potential of Microbial Strains, Used in Fermentation for Probiotic Food, Beverages and in Synbiotic Supplements, as Psychobiotics for Cognitive Treatment through Gut–Brain Signaling

**DOI:** 10.3390/microorganisms10091687

**Published:** 2022-08-23

**Authors:** Divakar Dahiya, Poonam Singh Nigam

**Affiliations:** 1 Wexham Park Hospital, Wexham Street, Slough SL2 4HL, UK; 2 Biomedical Sciences Research Institute, Ulster University, Coleraine BT52 1SA, UK

**Keywords:** probiotics, synbiotics, psychobiotics, food, beverage, nutrition, gut, brain, health, microbiota

## Abstract

Pure and viable strains of microorganisms identified and characterized as probiotic strains are used in the fermentation process to prepare probiotic food and beverages. These products are sources of nutrition and help in the maintenance of gut microflora. The intake of food products prepared with the use of probiotic microorganisms and containing their metabolites and whole microbial cells can be considered as a natural formulation of synbiotic products with prebiotic substrates and culture. Other than through the intake of fermented food and beverages, probiotic microorganisms can be taken through a supplement, which is a complementary form prepared by combining separate sources of prebiotic substrates and specific probiotic cultures. Whether a fermented solid food or beverage, both the components in the product are in a synergistic relationship and contribute to several health benefits at a lower cost. The aim of this article is to review the relevant literature and present the outcomes of recent studies which have been conducted to explore the clinical potential of probiotic strains and their effect on psychological conditions. Studies have shown the relationship between gut microbiota and the brain, and their interaction through signaling. The studies have concluded that the gut–brain axis can be manipulated with the intake of probiotic foods or synbiotic supplements containing specific probiotic strains accompanied with their complementary prebiotics for the enhanced sustainability of healthy GIT microflora.

## 1. Introduction

The constituents in the intake of food and nutrients affect the development of different microbial strains in the gastrointestinal tract (GIT). Hence, it is sensible to consider the fact that the nutritional and bioactive components could be the regulators and the main determinants of the gut microbiota composition. Research has confirmed that the gut microbiota significantly contribute to our general well-being, and their potential can also be explored to help in the remediation of psychological issues [1]. An improvement in the composition of gut microbiota can affect the intelligence, mood, behaviour, autism, and psychological well-being of the host through an important partnership between gut and brain [2]. There have been several studies on the composition of the gut microbiota and its effect on the health of its host. The studies were based on interventions using prebiotics and probiotics, mainly due to their multifaceted health benefits in many general disorders [3]. Beneficial gut microbiota developed due to the consumption of probiotic strains in the host’s gut, utilizing the prebiotics as their suitable substrates to increase and sustain their population in GIT [4,5]. The availability of prebiotics supports the growth and colonization of a large number of beneficial gut bacteria. The resident gut microflora also act toward the exclusion of various pathogenic bacteria, provide other health benefits such as immune-modulatory properties, and enhance the integrity of the gut barrier [6].

### 1.1. Eligibility of Microbial Strains as Probiotic Cultures

The definition of probiotics given by the Food and Agriculture Organization and the World Health Organization explains that the live microorganisms, when administered in adequate amounts, confer a health benefit on the host [7,8]. Probiotic cultures are mostly lactic acid bacteria, included under the category “Generally Recognized As Safe (GRAS)” by the US Food and Drug Administration [9,10].

Probiotics usually comprise bacteria, including Lactobacillus, Bifidobacterium, and Bacillus, although a few strains of yeast, Saccharomyces, have also been included in probiotic cultures. According to the consensus panel of the International Scientific Association for Probiotics and Prebiotics, probiotic practices are only delivered by some specific strains of a certain class of bacteria, for instance, *Lactobacillus casei* and *Bifidobacterium bifidum* [10]. However, for consideration as efficient probiotics, the microbial strains used in food fermentation or consumed as encapsulated freeze-dried cells in supplements must have clinically demonstrated their benefit to the host. The other properties making probiotics eligible for consumption include their role in the function of the immune system and training them to distinguish between good cultures and pathogens. Probiotic cultures also assist with the assimilation of fibers for the production of short-chain fatty acids for gut health benefits [11,12].

### 1.2. Clinical Potential of Probiotics

The gut microbiota is composed of diverse and variable combinations of several microbial strains. Healthy stability of gut microorganisms must necessarily be maintained to perform everyday functions related to physiological and metabolic activities and sustain immunity to prevent disease development. If the balance of the gut microbiota is disturbed for different reasons, it could initiate a number of health concerns and cause the progression of certain ailments [13,14]. Persistently disturbed microbiota might result in several ailments including a decline in cognitive abilities [15,16].

There are several scientific reports with evidence supporting the inclusion of probiotics in nutrition as a resource to gain other health benefits. The probiotic products confer a number of health-related benefits such as increasing GIT control and inhibiting infections, tumors, and diseases by stimulating immunity and improving vitamin B production [17]. Recent articles have mentioned that probiotic cultures have shown the capability to enhance the efficacy of the COVID-19 vaccine, probably due to their effective immunomodulating [18,19,20].

This review article is based on relevant information collected from 120 selected published reports. Appropriate references have been cited supporting all information presented in separate sections, e.g., on the contribution of probiotic microorganisms to gut health and their potential in the treatment of psychological diseases and improvement in cognitive skills. The information presented in this article is only based on those GRAS strains used in the food industry for the production of yogurt, fermented milk, fermented functional foods, and beverages. For vegans and persons allergic to dairy food products, probiotic culture/s in the form of formulated supplements can be taken to maintain healthy GIT microbiota. Freeze-dried preparations of live cells of probiotic cultures are available as commercial products and in different forms to cater to different requirements of consumers. The health benefits of probiotic microorganisms and their role in the management of specific psychological disorders are discussed further in Section 4.

## 2. Probiotic Microorganisms Used in Products for Health and Nutrition

Several reports have presented their outcome that the gut microbiota can be affected and influenced by the intake of suitable diets and supplements [21,22]. Results have confirmed that the gut microbiome can be manipulated by the intake of functional probiotic food [23,24]. Fermented foods prepared with probiotics are considered safe for consumption. Probiotic cultures are being used in food and health treatments and as additives in animal feed. The microbial strains widely used in the food industry are mostly lactic acid bacteria (LAB); these probiotic strains are easily available and accessible [25]. The characteristics of these cultures include the competitive ability to create a low pH with acid production (lactic acid) and other metabolites. All these microbial metabolites can play a role in the competition of LAB with other microorganisms during fermentation [26]. LAB have been broadly investigated for their beneficial properties in the food industry. Due to, their beneficial effects, some LAB strains have been given a QPS (Qualified Presumption of Safety) status in the EU for food applications. Though Lactobacillus and Lactococcus have achieved a GRAS status, Streptococcus and other species have received a status of GRAS/QPS [27]. The most commonly used probiotic bacteria for food applications belong to Lactobacillaceae family or Bifidobacterium genus [28,29].

### Probiotic Food and Beverage Products

A probiotic milk product, yogurt, is the best-known carrier for the efficient transfer of beneficial microbes into the gut [30,31]. Recently, the demand for a range of probiotic dairy products has intensified. Consumers are showing interest in products like fermented milk, Kefir, cheese, beverages prepared with a combination of probiotic strains, and synbiotic yogurt containing probiotics and prebiotic ingredients [32].

Fermentation by lactic acid bacteria also produces bactericidal proteins in dairy products, which are useful for the longer shelf life of these products [33,34]. Synbiotic yogurt has become a popular functional food that affects human health beneficially through the maintenance of good gut microflora [35,36]. Kefir fermented milk has increased consumers’ interest as it is available in form of a probiotic drink in portable bottles and it is easier and practical to consume anywhere on-the-go, in comparison to the consumption of thicker-consistency yogurt in non-resealable containers [37]. Cultures containing lactic acid bacteria are incorporated into milk for its fermentation; this process improves the bioavailability of nutrients and the sustainability of bacteria in fermented dairy products [38].

The consumption of products containing probiotics may provide an encouraging approach to the prevention of microbial dysbiosis [39]. The intake of synbiotic food products also influences the profiles of lipids and protects against irritable bowel syndrome (IBS), inflammatory bowel disease (IBD), and colorectal cancer, compared to the intake of either probiotics or prebiotics on their own [40]. In addition to LAB, *Saccharomyces boulardii* has been characterized as a probiotic yeast strain [41]. A bio-therapeutic process was developed based on the intake of *S. boulardii*. This probiotic strain was clinically tested through its oral administration to treat diarrhea developed after prolonged antibiotic treatment and recurrent illness associated with the infections caused by *Clostridium difficile* [42,43,44]. Different yogurt samples, containing this probiotic yeast added with varying concentrations of inulin as a complementary prebiotic, performed the safest as a physicochemical microbiological product with sensory properties, producing microrheology and microstructure [45,46]. The outcome of a research project reported the effect of fermented milk, produced using a probiotic strain of *Lactococcus lactis* ssp. *cremoris*, on the alleviation of infection caused by the invasion of intestinal epithelial cells by Salmonella [47]. Table 1 summarizes some of such studies on food and beverage products, prepared by use of characterized standard strains of probiotics.

## 3. Microbial Probiotic Strains in Synbiotic Supplements

It is not always possible to get the required numbers of live cultures available in the consumed portion size of fermented probiotic foods and beverages. For the requirement of sustaining a healthy gut microbiome, the resident population of GIT microflora can be improved by the intake of suitable probiotic supplements, containing the relevant viable strains and their required population, measured in the form of CFU. The formulated commercial products also indicate the number of probiotic cells and their dosage. Table 2 summarizes some of the commercially available supplements prepared using selected characterized strains of probiotic microorganisms and the complementary prebiotics added in their formulation.

## 4. Studies on the Clinical Potential of Probiotic Strains as Psychobiotics

Psychobiotics have been defined as probiotics that, when consumed, might produce benefits to mental health through the interactions of commensal gut bacteria with the brain [2]. It is important that probiotics should be accompanied by a suitable source of prebiotics, which enhance the growth of beneficial gut bacteria in the gut [1]. Prebiotics are natural substrates that produce specific changes in gut microflora and their activity after their assimilation into gut [73]. Prebiotic substrates like oligosaccharides are required for the growth of natural commensal bacteria. Most of the ingredients used as prebiotics, to test their effects, are oligosaccharides with up to nine saccharide units [74]. In synbiotic formulations, probiotics and prebiotics are used for their elaborate impacts on cognitive, emotional, and neural variables related to health and disease. A report has discussed the role of the gut-brain mechanism, which supports the clinical potential of probiotics to have psychobiotic effects, such as metabolite production [75].

These psychobiotics can produce positive psychiatric effects in psycho-pathology if taken through appropriate formulations and in correct quantities [76,77]. The bacteria most frequently used as probiotics have been the Gram-positive Bifidobacterium and from Lactobacillus families. These do not possess lipo-polysaccharide chains to cause pro-inflammatory effects and, as a result, their colonization in the gastrointestinal tract does not initiate immunological responses. In the presence of these bacteria, the immune system realizes the different activities of pro- and anti-inflammatory strains and, as a result, develops the appropriate responses through the detection of pro-inflammatory antigenic elements [78,79].

### 4.1. The Microbiome-Gut-Brain Axis

All microbial strains and their genomes inhabiting the GIT are included in the gut microbiome. Through the healthy and sustained colonization of the gut microbiome, a reciprocal gut-brain partnership develops, which is important for the collaborative efforts of the brain and gut in the maintenance of an individual’s health [80]. The system of the gut-brain axis is a complex procedure involving the microbiota in the gastrointestinal tract, connecting through the enteric nervous system to the brain [81]. Through the network of the nervous system, including the vagus nerve, the two-way communications between these entities regulate important the physiological functions of metabolism, digestion, assimilation, indulgence, immunity, and the equally important stress reactions [82,83,84].

The evidence was reported after a decisive study where mice were raised under sterile growth conditions to develop a complete absence of indigenous microflora. The microbe-free mice were found to be sensitive to stress and showed exaggerated physiological reactions compared to normally grown mice with a developed gut microflora. The unusual reactions toward the stress could be reverted through the development of probiotic-induced bacterial recolonization in initially germ-free mice [85]. This observation uncovered the gut microbiome’s participation in the development of hypothalamic–pituitary–adrenal axis. Other studies have also concluded that the gut microbiome participates in the regulation of important physiological processes, including the electrophysiological activity of the enteric nervous system [86], as well as immunomodulation and energy balance [87,88,89].

**Figure 1:**
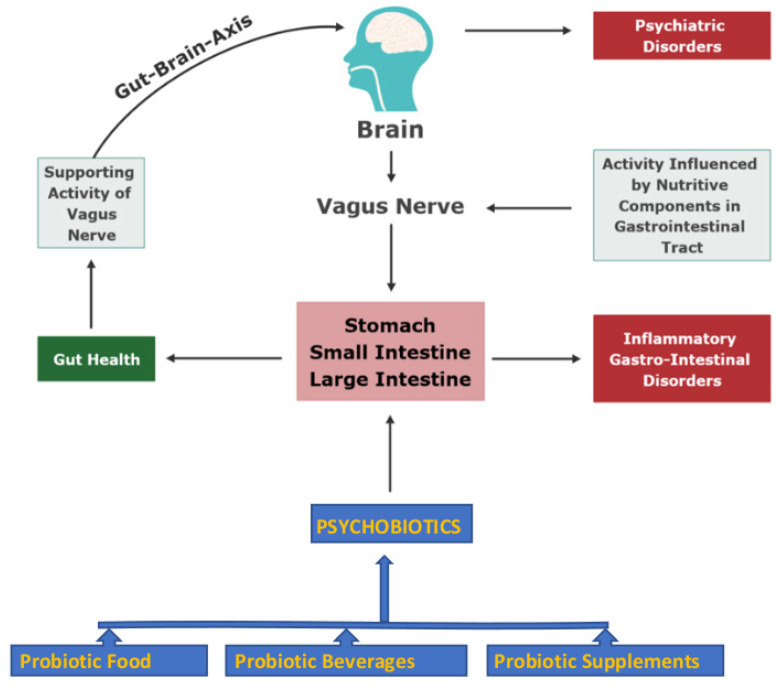
Psychobiotics in nutrition improve gut and brain health via the microbiome–gut–brain axis (Figure is drawn by authors Dahiya and Nigam). The consumption of probiotic products act as psychobiotics, where the microbiota-driven gut–brain axis reflects an interaction between the central nervous system and the intestine through the vagus nerve. Detailed information is available in references [1,2,3,4] for the boxes Probiotic food, Probiotic beverages, and Probiotic supplements. References [80,81,82,83,84,85,86,87,88,89] provide detailed information for the boxes Gut health, Inflammatory gastrointestinal disorders, Vagus nerve, Gut–brain axis, and Psychiatric disorders.

These reports have confirmed that psychobiotics are beneficial microbial strains, when dispensed and propagated in GIT supported by appropriate prebiotics, and influence the complex microbiome-brain relationship. The term should more precisely address psychobiotics as those synbiotics (probiotics + prebiotics), which can exert anti-anxiety results and act as an anti-depressant, distinguished through the changes in cognitive, emotional, and neural indicators [75]. Figure 1 shows the system of gut–microbiome-brain communication through the effects exerted by psychobiotics on the enteric nervous system and the immune system. Further information is required on dose responses and if there are any long-term effects to explore the clinical potential of psychobiotics.

### 4.2. Psychophysiological Effects of Psychobiotics

Psychobiotics affect psychophysiological markers of anxiety and depression. Most of the research on psychobiotics has been centered on rodent models, where studies have used behavioral tests and stress inductions to measure the levels of motivation, anxiety, and depression. The results of psychobiotic application to rodent models of neurodegeneration might also provide an experimental clinical understanding of human illnesses. Investigations with human volunteers represent a conclusive approach to the use of psychobiotics.

The psychophysiological effects of psychobiotics have been mainly analyzed in three ways. (1) Through the results gathered on cognitive and emotional processes. (2) Through the ystemic impacts on the hypothalamic–pituitary–adrenal–axis, glucocorticoid stress response, and the degree of inflammation indicated by irregular cytokine concentrations. (3) Through the effects measured through the observation of neural effects on neurotransmitters and proteins.

Pro-inflammatory cytokines have been reported for their positive association with depression and other psychiatric conditions [90]. In a study, the injection of interferon as a pro-inflammatory cytokine was shown to induce the condition of depression, which could be relieved through the action of antidepressants [91,92]. Appropriate neurotransmitters such as γ-aminobutyric acid (GABA) and glutamate [82,83,84] have been reported to control neural balance in excitation-inhibition. Proteins including brain-derived neurotrophic factors, important in learning and memory processes [93,94], are reduced in conditions of anxiety and depression, which could be reversed through the action of antidepressants [95].

The benefits noticed from the consumption of some probiotics were found to be similar to antidepressant effects. These findings were indicative of an earlier study, which used a mixture of two probiotic strains, *Lactobacillus rhamnosus* R0011 and *Lactobacillus helveticus* R0052, and produced results showing downregulation of hypothalamic–pituitary–adrenal axis activity [96].

In one study including in vivo research work, magnetic resonance spectroscopy [97] was used to study changes in central neurotransmitter concentrations induced by psychobiotics. Another study was conducted for a 4-week period administering the probiotic bacterial strain *Lactobacillus rhamnosus* JB-1 or a vehicle in healthy adult male mice. The results indicated raised concentrations of N-acetylaspartate glutamic acid, γ-aminobutyric acid, and glutamate in the probiotic-fed mice. Glutamate, the main excitatory neurotransmitter in the central nervous system, was found to be sensitive to probiotics [97]. This conclusion was reached after a general consensus pointed out that the ingestion of probiotics only results in their transient colonization in GIT [98].

### 4.3. Human Intervention Studies with Psychobiotic Supplements

In a research project, the impact of consumption of a probiotic milk was observed on the status of moods and cognition [99]; participants including males and females were given a milk beverage fermented with probiotic strain of *Lactobacillus casei* Shirota, and the comparison group received a placebo drink. The outcome of the intervention study was very interesting; participants that consumed probiotic drinks self-rated as happier rather than depressed, compared to those participants who consumed a placebo drink. Another study has provided evidence of improved mood in trials with a generally healthy group [100].

A randomized double-blind design project included male and female participants with no health problems who were given a test sample of probiotics consisting of two strains *Lactobacillus helveticus* and *Bifidobacterium longum* to consume over a period of 30 days; the comparison group received a placebo sample. A self-report completed by participants after the intervention period was based on a range of measures of mood and distress. Significant declines in negative mood and distress were reported by participants treated with probiotics compared to those who received a placebo supplement. These self-reported results were further confirmed by an analysis of cortisol levels [101]. The study also considered potential unfavorable effects, such as probiotic-induced losses or weakening in learning and memory. No evidence was produced of dysfunctions in learning and memory, suggesting a good safety profile for probiotics used as psychobiotics, without any consequential cognitive weakening.

In another randomized controlled project, studies were conducted over a period of 4 weeks of intervention. A group of 40 participants including healthy males and females was given a product for consumption containing a mixture of several probiotic strains, including *Bifidobacterium bifidum* W23, *B. lactis* W52, *Lactobacillus acidophilus* W37, *Lactobacillus brevis* W63, *Lactobacillus casei* W56, *Lactobacillus salivarius* W24, *Lactococcus lactis* W19, and *L. lactis* W58. Participants with probiotic treatment exhibited substantially reduced reactivity to sad moods, relative to participants who consumed a placebo supplement [102].

The cognitive enhancement effect of *Lactobacillus casei* Shirota has also been tested in a fascinating study with volunteers having academic stress prior to their assessment period. The study reported the consumption of milk fermented with *Lactobacillus casei* strain Shirota supported the preservation of the diversity of the gut microbiota, and it relieved the abdominal dysfunction in healthy medical students during the period of academic stress [103].

Another study was performed with a group of 44 student athletes, who were given a supplement containing the probiotic strain *Lactobacillus gasseri* OLL2809 LG2809. The performance was noticed as an elevation in their mood and a reduction in natural killer cell activity after vigorous physical exercises, relative to those students receiving a supplement of a placebo sample [104]. These results propose that the prescription of suitable probiotics might produce physiological benefits and have the potential to improve cognitive and mental performance in important activities of everyday life.

### 4.4. Performance of Specific Probiotic Strains in Psychobiotic Studies

Supplements containing prebiotics reduced the waking cortisol response and altered the emotional bias in healthy volunteers; prebiotic feeding elevated central-brain-derived neurotrophic factor, N-methyl-D-aspartate receptor subunits, and D-serine [105,106]. The role of Lactobacillus and Bifidobacterium was studied for symptom responses and relationships to cytokine profiles [107]. Some studies have demonstrated that only some specific strains produced the desired effects in psychobiotic studies in comparison to other probiotic strains, which either produced partial or no effect at all. Additionally, strains belonging to the same family may work differentially to affect the condition of anxiety. For example, in studies with rodent models, *Bifidobacterium longum* (B.)1714 was effective in reducing the depressive behavior in tail-suspension tests, although *Bifidobacterium breve* 1205 reduced anxiety in the tests of elevated-plus mazes [108].

Results of another study showed that *Bifidobacterium longum* (B.)1714 created desired cognitive advances, although the same results were not delivered by *Bifidobacterium breve* 1205 [109]. Despite the good performance achieved in all those studies using supplements, which contained a mixture of probiotic strains, the overall results from a specific strain could not be correctly concluded. It is possible that only some of the probiotic strains in the supplement mixture were producing the results, or the net performance was developed from the synergistic interactions between several strains used in the supplement mixture of strains [102,110,111].

Effects exerted by the strain *Lactobacillus helveticus* R0052 on inflammation and anxiety were reported to be associated with diet and genotype [112]. The adults of different age groups (young to elderly) have different microbiotic constructions developed over the years of life [113], and also the composition of microflora is not uniform between individuals of healthy aging and those aging with health issues [114]. The role of intestinal microbiota has been recognized as a part of a complex signaling network that connects to many systems modulating cognitive functions including learning, memory, and decision-making processes. In studies performed using both aging rodents and humans, administration of probiotic supplements reformed the gut microbiota; the population of Actinobacteria, Bacterioidetes, and Lactobacillus was found to increase, whereas *Clostridium difficile* concentrations decreased [111,115,116]. Modulation of intestinal microbiota by the probiotic helped in resetting the brain gene expression and ameliorated the age-related deficit in the archetypal model of plasticity and long-term potentiation. The data supported the notion that intestinal microbiota can be manipulated to study their positive impact on neuronal function [115].

### 4.5. Benefits and Limitations of Clinical Studies

Several studies performed in recent years have demonstrated that the normal gut microbiota modulates brain development and behavior; however, there are challenges in manipulating the gut microbiome for clinical studies [117]. Through efforts in managing the gut microbiome, how gut health influences the development and initiation of disease can be studied [118]. Although the role of the gut microbiota has been studied in the development, function, and disorders of the central nervous system, the enteric nervous system, and their benefits [119], there are obvious challenges in manipulating the gut microbiome that present limitations in exploring the potential of psychobiotics. Current animal studies are based on the use of forced oral feeding and gnotobiotic animals, which do not allow for non-invasive long-term access to the gut microbiome.

A recent research project has studied the development of a 3D-printed high-temperature resin cecal fistula implant in nine male Sprague–Dawley rats. The results present the most recent method of cecal fistula as an effective procedure that allows long-term and minimally invasive access to the gut microbiome. The feasibility of 3D-printed fistula implants through the body wall and into the cecum of rats to obtain long-term access to gut microbiome was evaluated [120]. The researchers analyzed the gut microbiome, vagal afferents in the hindbrain, and microglia activation. These analyses were used in ascertaining if the gut–brain neural axis was disrupted by fistula implantation. The results of determinations confirmed that, though the procedure induced a transient decrease in diversity in the gut microflora, that was resolved within a period of few weeks. Fistula implantation had no impact on food intake, body weight, fat mass, or microglia activation. There is certainly a scope for 3D-printed cecal fistula implantation to be used as an effective procedure. Such a device will allow long-term and minimally invasive access to gut microbiome in exploring the potential of psychobiotics.

## 5. Conclusions

In the last few decades, a number of studies have been performed on the effect of probiotics, beverages, and fermented foods as potential synbiotics on the gut microbiome. The interest of research projects has moved in the direction of clinical studies to identify how the GIT-microbiome could be manipulated for establishing and maintaining a healthy gut. The intake of food prepared with probiotic microbial strains has been recommended through the positive outcome of several studies that have reported that they have an effect on health and for the relief of a number of ailments. Probiotic food, fermented milk beverages, and synbiotic supplements have shown clinical potential for their use as psychobiotics. Several studies have proved their effectiveness in the performance of the gut–brain axis in psychiatric disorders. In conclusion, it is beneficial to explore the clinical potential of microbial strains, which are normally used in the preparation of probiotic food and beverages products or synbiotic supplements, as psychobiotics for cognitive treatment through gut–brain signalling-mechanism regulated by the vagus nerve system.

## Figures and Tables

**Table 1:** A list of characterized microbial strains used in fermentation for the preparation of probiotic food and beverage product.

No.	Strains of GRAS Microorganism	Probiotic Products	References
1.	*Lactobacillus casei*, *L. acidophilus*, *L. bulgaricus*, *Lactococcus lactis* *Bifidobacterium bifidum*, and *B. lactis* (*Bifidus actiregularis R*)	Probiotic commercial yogurts current information (accessed during April–July 2022)	[48]
2.	Food-grade *Lactococcus lactis* NZ3900	Fermented dairy product, a potential functional drink for oral vaccine delivery	[49]
3.	*Streptococcus thermophilus* and *Lactobacillus delbrueckii* spp. *bulgaricus* with cultures of *Leuconostoc lactis*	Fermented goat milk yogurt with enhanced sensory characteristics	[50,51]
4.	*Lactococcus lactis* ssp. cremoris strain JFR1	Fermented milk, effective in the Salmonella invasion of intestinal epithelial cells	[47]
5.	*Lactobacillus delbrueckii* spp. *Bulgaricus* and *Streptococcus thermophilus*	Yogurt with sensory characteristics, obtained in proto-synbiotic milk fermentation	[52]
6.	Monocultures of *Lactobacillus* (*Lb. acidophilus* La-5, *Lb. rhamnosus* K3, *Lb. plantarum* O20) and *Bifidobacterium* (*Bif. animalis* subsp. Lactis BB-12)	Fermented goat milk beverages with enhanced sensory characteristics	[53]
7.	Kefir grains of LAB with yeast cultures	Functional beverage Kefir	[21]
8.	*Lactiplantibacillus plantarum* and *Lactococcus lactis* subsp. *lactis*	For anti-clostridial activity during the cheese ripening process in the dairy industry	[54,55]
9.	*Lactobacillus casei* ATCC 393	Functional fermented milk	[22]
10.	Co-cultures of Probiotic Lactobacilli + *Streptococcus thermophilus*	Lactose-free fermented dairy beverages	[56]
11.	*Lactobacillus acidophilus* and *Bifidobacterium* sp.	Probiotic fortified yogurt for patients with lactose intolerance	[57]
12.	*Lactobacillus brevis*	Food additive lactic acid	[58]
13.	Kefir coculture lactococcus and lactobacillus	Feta-type cheese product	[59]
14.	*Lactobacillus casei* ATCC393	Functional Mizithra cheese	[60]
15.	*Lactobacillus casei* ATCC393 and *Lactobacillus bulgaricus* DSM20081 immobilized cells	Yogurts with enhanced probiotic viability and improved aromatic profile	[61]
16.	*L. paracasei* subsp. paracasei E6 and *L. paraplantarum* B1 and isolates from mature Melichloro cheese	Microencapsulated in biopolymer-based coacervate with enhanced cell viability for food products	[62]
17.	*Saccharomyces boulardii* CNCM I-745 (Probiotic yeast)	Probiotic and synbiotic yogurt with inulin with antioxidant and volatile capacities	[46]
18.	*Saccharomyces cerevisiae* DL6–20 and *Kluyveromyces marxianus* B13-5	Cheese with enhanced flavour and gamma-aminobutyric acid (GABA)	[63]

**Table 2:** A list of strains used in form of encapsulated freeze-dried cells for the formulation of supplement products [Information from online sources assessed during May–July 2022].

No.	Strains of Probiotic Microorganism	Products	Reference
1.	Clinically studied bacteria, including *Lactobacillus acidophilus* co-culture, *L. plantarum*, *Bacillus coagulans*, and Bifidobacterium co-culture (*B. animalis* subsp. *lactis*, *B. bifidum*)	Tribiotics for mind balance	[64]
2.	*Bacillus subtilis* PXN^®^ 21^®^	Supplement for the mind targeting cognitive function	[65]
3.	*Lactobacillus acidophillus*, *Bifidobacterium animalis* subsp. *Lactis*, *L*. *bulgaris*, and *L*. *salivarius*	Mega potency product acidophilus with pectin for gut health	[64]
4.	*Lactobacillus acidophilus*, *Lactobacillus gasseri*, *Bifidobacterium animalis* subsp.* lactis*, and *Bifidobacterium bifidum*	Good-gut live friendly bacteria with support for women’s health	[66]
5.	*Lactobacillus acidophilus, Bifidobacterium animalis* subsp.* lactis*, and *B. bifidum*	Good-gut live friendly bacteria with skin and hair support	[66]
6.	*Lactobacillus casei*, *L. acidophilus*, *L. bulgaricus*, *Lactococcus lactis*, *Streptococcus thermophilus*, *Bifidobacterium bifidum*, and *B. longum*	Supporting gut healthand gut microbiome	[67]
7.	*Lactobacillus paracasei*, *Lactobacillus reuteri*, and *Lactobacillus rhamnosus*	Optibac intimate flora for women, scientifically proven to reach the intimate area.	[66]
8.	*Bacillus subtilis*, *Bifidobacterium bifidum*, *B. breve*, *B. infantis*, *B. longum*, *Lactobacillus acidophilus*, *L. delbrueckii* ssp.* bulgaricus*, *L. casei*, *L. plantarum*, *L. rhamnosus*, *L. helveticus*, *L. salivarius, Lactococcus lactis* ssp.* lactis*, and *Streptococcus thermophilus*	Multi-strain advanced formulation for the digestive system	[65]
9.	Fourteen strains of live bacteria	Bio-Kult Migréa for head discomfort. Advanced multi-action formulation	[65]
10.	Six live strains with a prebiotic	Bloating relief and gut health support	[68]
11.	Five strains including *L. acidophilus* NCFM^®^	A premium probiotic support for digestion, immunity, and energy	[69]
12.	Thirty clinically studied broad-spectrum strains	Steady-state and targeted controlled release of probiotic cells in gut	[70]
13.	Sixteen strains of friendly bacteria: including *Bifidobacterium*, *Lactobacillus*, and *Streptococcus*	Active cultures for gut flora	[71]
14.	Proprietary bacterial blend (*Lactobacillus acidophilus*, *Streptococcus thermophilus*, *L*. *rhamnosus*, *L*. *Salivarious*, and *Bifidobacterium bifidum*)	Balance gut health with good bacteria to balance out the bad bacteria	[72]

## Data Availability

Not applicable.
